# The extended regulatory networks of SXT/R391 integrative and conjugative elements and IncA/C conjugative plasmids

**DOI:** 10.3389/fmicb.2015.00837

**Published:** 2015-08-20

**Authors:** Dominic Poulin-Laprade, Nicolas Carraro, Vincent Burrus

**Affiliations:** Laboratory of Bacterial Molecular Genetics, Département de Biologie, Faculté des Sciences, Université de Sherbrooke, Sherbrooke, QC, Canada

**Keywords:** SXT/R391, IncA/C, SGI1, regulation, integrative and conjugative elements, conjugative plasmids, genomic islands, pVCR94

## Abstract

Nowadays, healthcare systems are challenged by a major worldwide drug resistance crisis caused by the massive and rapid dissemination of antibiotic resistance genes and associated emergence of multidrug resistant pathogenic bacteria, in both clinical and environmental settings. Conjugation is the main driving force of gene transfer among microorganisms. This mechanism of horizontal gene transfer mediates the translocation of large DNA fragments between two bacterial cells in direct contact. Integrative and conjugative elements (ICEs) of the SXT/R391 family (SRIs) and IncA/C conjugative plasmids (ACPs) are responsible for the dissemination of a broad spectrum of antibiotic resistance genes among diverse species of *Enterobacteriaceae* and *Vibrionaceae*. The biology, diversity, prevalence and distribution of these two families of conjugative elements have been the subject of extensive studies for the past 15 years. Recently, the transcriptional regulators that govern their dissemination through the expression of ICE- or plasmid-encoded transfer genes have been described. Unrelated repressors control the activation of conjugation by preventing the expression of two related master activator complexes in both types of elements, i.e., SetCD in SXT/R391 ICEs and AcaCD in IncA/C plasmids. Finally, in addition to activating ICE- or plasmid-borne genes, these master activators have been shown to specifically activate phylogenetically unrelated mobilizable genomic islands (MGIs) that also disseminate antibiotic resistance genes and other adaptive traits among a plethora of pathogens such as *Vibrio cholerae* and *Salmonella enterica*.

## Mobile Genetic Elements in the Modern World of Multiresistance

The discovery of penicillin by Alexander Fleming over 80 years ago marked the end of the pre-antibiotic era and revolutionized the prevention and treatment of many bacterial infections responsible for high morbidity and mortality. However, Sir Fleming himself warned the scientific community about antibiotic resistance and foresaw that inadequate usage of antibiotics could lead to “educated microbes.” Since then, the use and misuse of antibiotics have led to the rapid and widespread emergence and selection of microorganisms resistant to a wide range of antimicrobial compounds. Today, multidrug resistance (MDR) has become one of the most alarming healthcare issue on a global scale, so much so that in 2014 the World Health Organization (WHO) predicted a bleak short-term future: “A post-antibiotic era—in which common infections and minor injuries can kill—far from being an apocalyptic fantasy, is instead a very real possibility for the 21st Century” (World Health Organization, 2014).

Point mutations and/or gene amplification can allow bacteria to withstand hostile environments, such as the exposure to antimicrobial compounds (Gorgani et al., 2009; Davies and Davies, 2010; Toprak et al., 2012). Most often, MDR results from the acquisition by horizontal gene transfer of mobile genetic elements carrying multiple antibiotic resistance genes (Burrus et al., 2006; Mulvey et al., 2006; Welch et al., 2007; Escudero et al., 2014). Conjugation, which mediates DNA transfer between two bacterial cells in direct contact, is the most effective mechanism of horizontal gene transfer in terms of host range and quantity of genes translocated to a recipient cell per transfer event (Llosa et al., 2002; de la Cruz et al., 2010). Integrative and conjugative elements (ICEs) and conjugative plasmids of various incompatibility groups were shown to have a major impact on the global emergence of multidrug resistant pathogenic bacteria, in both clinical and environmental settings (Burrus et al., 2006; Fricke et al., 2009; Smillie et al., 2010; Wozniak and Waldor, 2010; Guglielmini et al., 2011; Walsh et al., 2011; Carattoli, 2013). Although both types of elements transfer from cell to cell by conjugation, their mechanism of persistence in the bacterial host cell genome is different. On the one hand, ICEs maintain themselves by integration into the chromosome of their host and excise prior to transfer as circular molecules (Burrus et al., 2002; Burrus and Waldor, 2004; Wozniak and Waldor, 2010). On the other hand, conjugative plasmids are maintained by replication as episomes, i.e., DNA molecules that are distinct from the chromosome.

This review focuses on the regulatory networks that govern the conjugative transfer of ICEs belonging to the SXT/R391 family (SRIs) and conjugative plasmids of the A/C incompatibility group (ACPs). Both classes of elements bear highly similar and nearly syntenic core sets of conserved genes and code for comparable transfer activator complexes (Wozniak et al., 2009; Carraro et al., 2014a; Poulin-Laprade et al., 2015). Recent investigations of the regulatory circuitries that activate SRIs and ACPs transfer have also contributed to the discovery of three classes of genomic islands (GIs) specifically mobilized by either SRIs or ACPs (Doublet et al., 2005; Daccord et al., 2010, 2012; Carraro et al., 2014a; Poulin-Laprade et al., 2015).

## Diversity and Prevalence of SRIs and ACPs

SRIs and ACPs are major contributors to worldwide dissemination of adaptative traits such as antibiotic resistance among several species of *Enterobacteriaceae* and *Vibrionaceae* of clinical origin or isolated from the aquatic environment.

The SXT/R391 family is one of the largest, diverse and well-studied set of ICEs among Gram-negative bacteria. Extensive experimental and bioinformatic studies have led to a deeper understanding of their prevalence, diversity, and evolution (Boltner et al., 2002; Wozniak et al., 2009; Garriss and Burrus, 2013; Carraro and Burrus, 2014; Spagnoletti et al., 2014). SRIs are large conjugative elements (79 to 110 kb) found integrated into the 5′ end of *prfC* in the chromosome of several species of *Vibrio*, *Photobacterium*, *Providencia*, *Proteus*, *Alteromonas*, *Marinomonas*, and *Shewanella*, and are easily transferred to *E. coli* in the laboratory (Coetzee et al., 1972; Waldor et al., 1996; Hochhut and Waldor, 1999; Beaber et al., 2002a; Pembroke and Piterina, 2006; Osorio et al., 2008; Harada et al., 2010; Rodriguez-Blanco et al., 2012; Badhai et al., 2013; Lopez-Perez et al., 2013; Spagnoletti et al., 2014). Notably, SRIs played a key role in the dissemination of MDR in the seventh-pandemic lineage of *V. cholerae*, the etiological agent of the diarrhoeal disease cholera (Spagnoletti et al., 2014). *V. cholerae* is endemic in Asia, Africa, and Central America and epidemics of cholera are usually blooming in locations where the sanitation infrastructures and access to clean water are compromised. Indeed, cholera is considered by the WHO as an indicator of sanitation mismanagement and humanitarian crisis (e.g., refugee camps). Currently, most clinical isolates of *V. cholerae* carry an SRI and are multidrug resistant worldwide. Most SRIs found in epidemic strains of *V. cholerae* contain the genes *floR*, *strBA*, *sul2*, and *dfrA1* or *dfr18*, respectively conferring resistance to florfenicol/chloramphenicol, streptomycin, sulfamethoxazole and trimethoprim (Waldor et al., 1996; Hochhut et al., 2001; Wozniak et al., 2009). Sulfamethoxazole and trimethoprim have synergistic antibacterial activities and are often used in combination for the treatment of cholera (Kaper et al., 1995). Other SRIs from the aquatic environment and from diverse pathogens confer resistance to kanamycin (*aph*) or tetracycline (*tetAR*) (Coetzee et al., 1972; Osorio et al., 2008; Wozniak et al., 2009; Bi et al., 2012). In the countries where the sanitation infrastructures are appropriate, the domestic cases of cholera and other vibriosis caused by hosts of SRIs are widely associated with the consumption of raw or undercooked seafood (Morris, 2003; Song et al., 2013; Hara-Kudo and Kumagai, 2014; Robert-Pillot et al., 2014). For instance, a few cases of cholera acquired in the US are declared each year. These sporadic cholera cases are generally attributed to consumption of seafood gathered from the US Gulf coast (Loharikar et al., 2015). Antibiotic resistance genes carried by SRIs are also troublesome for aquaculture as resistance genes can hinder the treatment of diseased fish and enter the food chain (Osorio et al., 2008; Rodriguez-Blanco et al., 2012; Nonaka et al., 2014). Indeed, consumption of raw fish and shellfish contaminated by live bacteria bearing SRIs could facilitate the dissemination of MDR among *Gammaproteobacteria* of the human host microbiome.

ACPs are large (>110 kb) circular plasmids grouped as a family based on the high percentage of sequence conservation of their *repA* gene, which codes for their replication intiator protein (Llanes et al., 1994, 1996; Carattoli et al., 2005; Fricke et al., 2009). Multidrug resistant ACPs are found worldwide in pathogens associated with human infections such as *Citrobacter freundii*, *V. cholerae*, *Salmonella enterica*, *Proteus mirabilis*, *E. coli*, *Yersinia pestis* and *ruckeri*, *Klebsiella pneumoniae*, and *Providencia stuartii* (Bauernfeind et al., 1996; Galimand et al., 1997; Giles et al., 2004; Welch et al., 2007; Ding et al., 2008; Fricke et al., 2009; Call et al., 2010; Fernandez-Alarcon et al., 2011; Lindsey et al., 2011; Walsh et al., 2011; Carattoli, 2013; Carraro et al., 2014b; Rahman et al., 2014). ACPs carrying MDR are also increasingly encountered in enteropathogenic bacteria recovered from food-producing animals and food products, mainly *S. enterica* and *E. coli* (Glenn et al., 2011; Lindsey et al., 2011; Randall et al., 2011; Folster et al., 2012; Del Castillo et al., 2013; Guo et al., 2014). Disturbingly, recent studies identified multiple extended-spectrum β-lactamases (ESBLs)-encoding ACPs conferring resistance to a wide range of β-lactam antimicrobials (Fernandez-Alarcon et al., 2011; Folster et al., 2011, 2012; Walsh et al., 2011; Harmer and Hall, 2015). Carbapenems were the last effective β-lactams for the treatment of infectious bacteria carrying ESBLs. Unfortunately, several recently isolated ACPs propagate the infamous New Delhi metallo-β-lactamase *bla*_*NDM-1*_ gene and its variants, which code for zinc metallo-β-lactamases that hydrolyze all penicillins, cephalosporins and carbapenems (Walsh et al., 2005, 2011; Yong et al., 2009; Nordmann et al., 2011; Tijet et al., 2015).

ACPs and SRIs are a threat to antibiotic therapies due to the large variety of antibiotic resistance genes that they bear on dynamic genetic structures such as integrons and transposons, further promoting the exchange and capture of resistance genes from other mobile genetic elements (Hochhut et al., 2001; Mazel, 2006; Welch et al., 2007; Fricke et al., 2009; Wozniak et al., 2009; Lindsey et al., 2011; Carraro et al., 2014b). Acquisition and exchange of antibiotic resistance genes are strongly enhanced by the broad host range of these elements, which can easily spread across several genera and species of *Gammaproteobacteria*. This phenomenon is likely further exacerbated by their mechanism of transfer as single-stranded DNA molecules have been shown to stimulate the SOS response in recipient cells, thereby promoting the intra- and inter-integrons movement of resistance cassettes (Guerin et al., 2009; Baharoglu et al., 2010, 2012; Cambray et al., 2011; Escudero et al., 2014).

## Modular Organization of SRIs and ACPs

**FIGURE 1:**
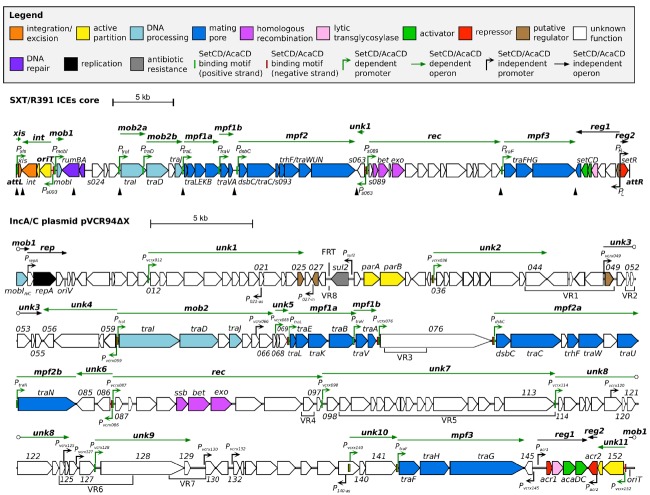
**Schematic representation of the genetic organization and transcriptional units of the conserved core of SXT/R391 ICEs (integrated in ***prfC***) and pVCR94ΔX (circular map linearized at the start position of gene ***mobI***) adapted from Poulin-Laprade et al. (2015) and Carraro et al. (2014a, 2015a).** Genes are represented by arrows and color coded according to their function as indicated in the legend. For clarity, ORF names *vcrxXXX* were shortened as *XXX* for pVCR94ΔX. SetCD- and AcaCD-binding motifs located on positive and negative DNA strands are represented by light green and red narrow boxes, respectively. Operons are indicated by arrows positioned above represented genes. SetCD- and AcaCD-regulated promoters and operons are colored in green. Open circles mark operons interruptions generated by the map format. *mob1-2*, DNA processing; *rep*, replication; *unk1-11*, unknown; *mpf1-3*, mating pore formation; *rec*, recombination; *reg1-2*, regulation. *P*_021–*as*_ and *P*_140–*as*_: *vcrx021* and *vcrx140* antisens promoters, respectively. *P*_027–*in*_: *vcrx027* internal promoter. Black triangles show the position of variable cargo DNA in SRIs, while variable DNA regions inserted in the conserved core of ACPs are indicated below genes (VR1 to VR8). The origin of replication (*oriV*) and the origin of transfer (*oriT*) are indicated. The position of the FRT site resulting from the deletion of the antibiotic resistance gene cluster in pVCR94 is also shown.

All SRIs share 47 kb of DNA corresponding to a highly conserved core set of 52 genes with over 95% identity at the nucleotide level (Wozniak et al., 2009). About half of these genes have been shown to be essential to ensure the basic maintenance, transfer and regulatory functions of SRIs. These essential genes are clustered in four main modules (Figure 1), i.e., the *int* module which codes for the integrase and excisionase and ensures intracellular mobility, the *mob* and *mpf* modules which code for a type IV secretion system (T4SS) and is responsible of the intercellular mobility (DNA processing and mating pore formation), and the *reg* module coding for the regulatory network governing the expression of the other modules. Each module can contain one to several transcriptional unit(s) (Figure 1; Poulin-Laprade et al., 2015). The *reg* module of SRIs is the most highly conserved locus amongst members of this family of ICEs (Wozniak et al., 2009).

ACPs are characterized by ∼110 kb of conserved core genes with over 98% nucleotide sequence identity (Fricke et al., 2009; Fernandez-Alarcon et al., 2011; Del Castillo et al., 2013; Carraro et al., 2014b; Harmer and Hall, 2014, 2015). Although ACP conserved core is larger than the one shared by SRIs, their organization is highly similar and syntenic (Welch et al., 2007; Wozniak et al., 2009). In particular, the *tra* genes of the *mob* and *mpf* modules of ACPs and SRIs are reminiscent of the IncFI F and IncHI1 R27 plasmids suggesting a common ancestry (Lawley et al., 2003). One of the most striking differences between SRIs and ACPs reflects their respective biology. The *int* module, which ensures chromosomal integration and excision of SRIs, is replaced by the *rep* module driving the replication of the episomal ACPs. The conserved core of ACPs also contains several genes of unknown functions beyond those also found in SRIs.

Distinctive features of the individual members of SRI and ACP families are provided by insertions of variable cargo DNA in hotposts dispersed in their respective conserved core. These insertions vary in size (from ∼60 to 20,000 bp) and encode adaptative traits that may provide a selective advantage to the bacterial host in specific conditions, such as resistance to antibiotics, heavy metals or phage infection, or synthesis of the second messenger c-di-GMP (Welch et al., 2007; Fricke et al., 2009; Wozniak et al., 2009; Bordeleau et al., 2010; Carraro et al., 2014a).

## Control of the Conjugative Functions of SRIs and ACPs

Control of SRI and ACP conjugative transfer is a key attribute for their propagation and stability. Excessive repression would impair their dissemination, while overactivation would be a burden for the bacterial host causing reduced fitness, and ultimately their instability in the cell population (Lundquist and Levin, 1986; Scott et al., 1988; Beaber et al., 2002b; Ramsay et al., 2006; Bellanger et al., 2009; Haft et al., 2009). Moreover, SRIs and ACPs not only drive their self-transfer, but also the transfer of phylogenetically unrelated mobilizable genomic islands (MGIs). Additionally, SRIs in association with MGIs can mobilize up to 1.5 Mb of chromosomal DNA each in Hfr-like conjugal events initiated prior to their excision (Hochhut et al., 2000; Daccord et al., 2010). Hence, these elements can potentially mobilize more than 60% of *V. cholerae* chromosome I in a single conjugal event.

Transcriptional repressors encoded by SRIs and ACPs repress the expression of master activator genes, maintaining these elements in a quiescent state in most cells of the bacterial population. Both SRIs and ACPs thrive in a large array of *Enterobacteriaceae* and *Vibrionaceae*, which implies that their regulatory networks are likely autonomous and orthogonal, i.e., they allow the activation/repression of the element while avoiding crosstalks with regulatory networks of the host cell.

### The Regulation Module of SRIs and ACPs

**FIGURE 2:**
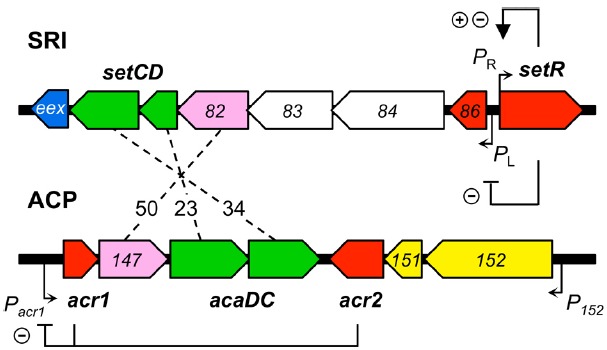
**Comparison of the regulatory modules of SXT/R391 ICEs (SRI) and IncA/C plasmids (ACP).** The genes are color-coded as indicated in Figure 1 legend. Numbers between the elements represent the percentage of identity between orthologous proteins. The regulation exerted by SetR, Acr1, and Acr2 is indicated (minus sign for repression, plus sign for activation). For clarity, ORF names *s0XX* were shortened as *XX* for SRI and *vcrxXXX* as *XXX* for ACP.

SRIs and ACPs bear distinct regulatory modules that govern their self-transmissibility (Figure 2). These regulatory modules code for unrelated repressors: SetR for SRIs and Acr1 and Acr2 for ACPs (Beaber et al., 2004; Carraro et al., 2014a). In contrast, the regulatory module of SRIs and ACPs code for related transcriptional activator complexes, respectively SetCD and AcaCD, that drive the expression of the conjugative genes and other functions (Beaber et al., 2002b; Carraro et al., 2014a; Poulin-Laprade et al., 2015). SetCD and AcaCD are distant relatives of FlhCD, the master activator of flagellum biosynthesis in many Gram-negative bacteria (Chevance and Hughes, 2008; Fitzgerald et al., 2014). Recent studies established the AcaCD and SetCD regulons and refined the models of transcriptional organization of the functional core of both types of elements (Figure 1; Carraro et al., 2014a; Poulin-Laprade et al., 2015).

### Repression of SRIs Dissemination

#### The SetR Repressor

The dominant regulatory state of SRIs is the quiescent state in which the element is integrated into the chromosome and the genes associated with recombination and transfer are silent (Beaber et al., 2004; Poulin-Laprade et al., 2015). In this dormant state, very few genes are transcribed, including genes independently regulated belonging to cargo DNA (e.g., antibiotic resistance genes) and *setR*. The *setR* gene is located at the rightmost end of the integrated ICE (Figure 1). SetR is an acronym for SXT excision and transfer repressor. *setR* mRNA transcript is leaderless, expressed from the *P_R_* promoter, and codes for a 215-amino acid residue protein with a DNA binding helix-turn-helix motif (HTH_3, PF01381) in its N-terminal moiety and a C-terminal LexA-like autoproteolysis motif (Peptidase_S24, PF00717). SetR shares homology with λ CI-like repressors encoded by lambdoid bacteriophages (Beaber et al., 2002b). The pivotal role of SetR in SRIs regulation is reflected by the inability to generate a *setR* mutant of SXT without simultaneous *setR trans*-complementation or a preexisting *setCD* inactivation (Beaber et al., 2002b, 2004).

#### SetR Regulation of the *P_L_* and *P_R_* Early Promoters

SetR maintains the quiescent integrated state of SRIs by binding to four operator sites (*OL*, *O1*, *O2*, and *O3*) distributed in the intergenic region between *s086* and *setR* (Figure 2; Beaber and Waldor, 2004). Footprint assays revealed that the relative affinity of SetR for its operators is *O1* > *O2*≈*O3* > *OL* (Beaber and Waldor, 2004). SetR operator sites bear partial dyad symmetry and are separated by AT-rich spacers. An additional site located 800 bp downstream of the *P_L_* promoter was suggested but never assessed (Beaber and Waldor, 2004). It has been proposed that binding of SetR to the four operators between *s086* and *setR* leads to SetR’s autoregulation of the *P_R_* promoter (Beaber et al., 2004). Binding of SetR to *O1* is thought to lead to activation of the *P_R_* promoter. When the cellular pool of SetR exceeds a threshold, SetR is thought to repress its own expression by further binding to the low affinity *O3* operator, concealing the -10 element of the *P_R_* promoter (Beaber and Waldor, 2004). Beaber and Waldor (2004) observed the repressive effect of SetR on *P_R_* by monitoring the β-galactosidase activity of a *P_R_*-*lacZ* transcriptional fusion in strains containing or lacking SXT, or its Δ*setCD* or Δ*setCD* Δ*setR* mutants. Quantification of the β-galactosidase activity in these strains showed that the presence of SXT lowered the activity of *P_R_* by 30% (SXT^–^ versus SXT^+^) (Beaber and Waldor, 2004). Deletion of *setCD* did not significantly alter *P_R_* activity compared to the SXT^+^ background, whereas in cells containing SXT Δ*setCD* Δ*setR*, *P_R_* activity was comparable to cells lacking SXT, thereby confirming that SetR represses *P_R_* (Beaber and Waldor, 2004). SetR binding to *O1* and *OL* obstructs the *P_L_* promoter which drives *setCD* expression and subsequent activation of conjugative functions.

The mRNA transcript starting at *P_L_* codes for seven proteins including a predicted λ Cro-like repressor, the two subunits of the activator complex SetCD, and the entry exclusion determinant Eex (Figure 2; Beaber et al., 2002b; Beaber and Waldor, 2004; Marrero and Waldor, 2005; Poulin-Laprade et al., 2015).

#### Alleviation of SetR Repression

In donor cells, the inductive cue triggering SRI propagation is linked to the SOS response (Waldor et al., 1996; Beaber et al., 2004). Using the energy of ATP, RecA polymerizes onto single-stranded DNA, generating RecA-ssDNA filaments (RecA*) that are competent for homologous recombination and are also allosteric effectors unleashing the latent proteolytic activity of LexA and λ CI-like repressors (Little, 1984; Chen et al., 2008). Thus, RecA is the central factor linking DNA damages (sometimes caused by antibiotics) to the cellular SOS stress response (DNA mutagenesis and repair), and to the induction of conjugative transfer of SRIs that are major vectors of MDR (Beaber et al., 2004; Baharoglu et al., 2010). Inspired by the extensive work done on the λ CI repressor, the link between RecA* and SetR was drawn with the mutant *setR*^G49E^ in which the Ala-Gly cleavage site activated by RecA is disrupted (Gimble and Sauer, 1985; Beaber et al., 2004). As expected, the *setR*^G49E^ mutant of SXT is unresponsive to mitomycin C, a DNA damaging agent known to trigger the bacterial SOS response.

Upon DNA damage, SetR becomes a substrate for RecA*-mediated self-cleavage, thereby alleviating SetR’s repression on *P_L_* and allowing *setCD* expression. The -10 and -35 promoter elements of *P_L_* are more similar to the recognition motif of σ^70^-bound RNA polymerase (RNAP) than those of *P_R_*, likely leading to a quicker isomerization into an open complex competent for transcription initiation. Alleviation of SetR repression would then be sufficient for recognition of *P_L_* by RNAP, without the need of a transcriptional activator. This model is reminiscent of the regulation of λ *P_R_* and *P_RM_* early promoters (Strainic et al., 2000; Ptashne, 2004).

SetR acts as a sentinel “sensing” DNA damages and triggering the “escape” of SRIs to recipient cells. For an optimal responsiveness and avoidance of cellular resources misallocation, SetR expression is tightly regulated and maintained at low levels (Beaber and Waldor, 2004). The *setR* transcript is a leaderless mRNA; the absence of a Shine-Dalgarno sequence is a post-transcriptional mechanism that likely contributes to a low intracellular level of SetR protein (Van Etten and Janssen, 1998; Beaber and Waldor, 2004). Spontaneous induction of the SOS response in a subpopulation of cells is thought to account for the low basal transfer of SRIs, which varies between individual SRIs for reasons that remain unknown (Beaber and Waldor, 2004; McCool et al., 2004; McGrath et al., 2005; Poulin-Laprade et al., 2015).

### Repression of ACPs

While no SetR homolog has been found in ACPs, their regulatory module codes for two repressors named Acr1 and Acr2 (IncA/C repressor 1 and 2; Carraro et al., 2014a). *acr1* codes for a 90-amino acid Ner-like protein that is mainly composed of a helix-turn-helix DNA binding domain (HTH_35, PF13693). Acr1 directly represses its own expression from the constitutive promoter *P_acr1_* (Figure 2). This promoter drives the expression of *acr1* and also the expression of *acaC* and *acaD*, which code for the activator complex AcaCD. Acr2 is a 139-amino acid H-NS-like repressor (Histone_HNS, PF00816) that also directly represses *P_acr1_* (Carraro et al., 2014a,b). H-NS proteins are known to globally repress expression of horizontally acquired DNA by binding AT-rich sequences (Dorman, 2004, 2014; Navarre et al., 2006). Besides *P_acr1_*, Acr2 might also repress other plasmid- or host-borne promoters, potentially having a wider impact on the biology of ACPs and their interaction with host cells.

The frequency of transfer of ACPs varies widely from non detectable to very high (1 in 10 cells for pVCR94; Welch et al., 2007; Fricke et al., 2009; Carraro et al., 2014b). Inducing factors triggering the conjugative transfer of ACPs have yet to be identified (Carraro et al., 2014a,b). Consistent with the absence of SOS-dependent repressors such as λ CI or ImmR, conjugative transfer of ACPs is independent of *recA* and the SOS response (Auchtung et al., 2005; Carraro et al., 2014b).

### The Heteromeric Complexes SetCD and AcaCD

It was previously established that individual deletion of either *setC* or *setD* abolished the excision and transfer of the prototypical SRI SXT (Beaber et al., 2002b). These deletions were complemented in *trans* with plasmids expressing the individual genes, thereby confirming the central role of SetC and SetD in the biology of SRIs. Transcriptional *lacZ* fusions with promoters driving the expression of *int*, *traL* and *traG* demonstrated that SetCD is a transcriptional activator of the site-specific recombination and conjugative transfer genes (Beaber et al., 2002b; Poulin-Laprade et al., 2015).

A similar characterization was recently carried out for *acaC* and *acaD*, which code for the master activator of ACP conjugative transfer (Carraro et al., 2014a). For both sets of transcriptional activators, genetic assays strongly suggest that the products of the *setD*-*setC* and *acaD*-*acaC* genes assemble into higher order protein complexes designated SetCD and AcaCD, respectively. While no direct experimental evidence support the oligomerization of SetCD, AcaD was shown to copurify with 6xHis-tagged AcaC subunit, supporting the formation of heteromeric complexes as observed for the flagellar gene activator complex FlhCD (Wang et al., 2006; Carraro et al., 2014a).

Conflicting evidence suggest a possible autoregulation of SetCD expression. On the one hand, overexpression of SetCD was reported to result in a 40-fold activation of expression of a chromosomal *setD::lacZ* fusion in SXT (Beaber et al., 2002b). On the other hand, expression from *P_L_*, which drives *setCD* expression, remained unaffected by deletion of *setCD* regardless of the presence of mitomycin C (Beaber et al., 2004). An exhaustive list of the promoters targeted by SetCD was recently established for three representative members of the SRI family (SXT, R391 and ICE*Vfl*Ind1) using chromatin immunoprecipitation coupled with exonuclease digestion (ChIP-exo) and RNA sequencing (RNA-seq; Poulin-Laprade et al., 2015). No SetCD binding site was found upstream of *P_L_* or elsewhere in the regulatory module.

**FIGURE 3:**
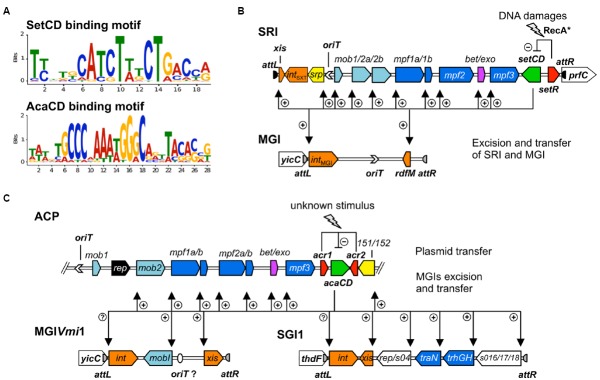
**Activation by heteromeric complexes SetCD and AcaCD. (A)** Experimentally determined recognition motifs of SetCD and AcaCD (Carraro et al., 2014a; Poulin-Laprade et al., 2015). **(B)** Representation of SetCD targets in SRIs and in MGIs they mobilize. The arrows indicate transcriptional repression by SetR (minus sign) and transcriptional activation by SetCD (plus signs). **(C)** Representation of AcaCD targets in ACPs and in MGIs from MGI*Vmi*1 and SGI1 families. The arrows indicate transcriptional repression by Acr1 and Acr2 (minus sign) and transcriptional activation by AcaCD (plus signs). In both B and C panels, the modules of DNA processing (*mob*) and mating pair formation (*mpf*) are indicated and color-coded as described in Figure 1 legend.

A similar experimental approach also allowed to establish the list of the promoters targeted by AcaCD in pVCR94ΔX, a prototypical ACP lacking most of its resistance genes (Carraro et al., 2014a,b). The DNA motifs recognized by SetCD and AcaCD were deduced from the multiple targets that were experimentally determined. Operator sites for SetCD and AcaCD fixation greatly differ from each other, and from the DNA motif recognized by *E. coli* FlhCD (Figure 3A; Carraro et al., 2014a; Fitzgerald et al., 2014; Poulin-Laprade et al., 2015). Despite their functional homology, SetCD, AcaCD, and FlhCD exhibit a high degree of divergence, which is reflected in their respective DNA target preference and specificity (Liu and Matsumura, 1994; Carraro et al., 2014a; Fitzgerald et al., 2014; Poulin-Laprade et al., 2015).

### SetCD and AcaCD are Pleiotropic Transcriptional Activators

In many mobile genetic elements, genes involved in a given biological function are often arranged in an operon structure within a single module expressed from a single promoter (Celli and Trieu-Cuot, 1998; Toussaint and Merlin, 2002; Auchtung et al., 2005; Carraro et al., 2011). The genes coding for the conjugative machinery of the *E. coli* IncF1 F plasmid or the *Enterococcus faecalis* ICE Tn*916* are good examples of such an organization (Celli and Trieu-Cuot, 1998; Lawley et al., 2003). In contrast, the conjugation modules of SRIs and ACPs are fragmented in multiple and distinct operons (Figures 1 and 3B,C). This fragmentation of functional modules is most often attributed to insertions of variable cargo DNA, insertion sequences (IS) and transposons (Fricke et al., 2009; Wozniak et al., 2009; Fernandez-Alarcon et al., 2011; Meinersmann et al., 2013). These insertions occur in sites most likely selected because of their minimal impact on genes essential for transfer and subsequent maintenance of SRIs and ACPs in bacterial populations. Discontinuity of the functional modules complexifies the genetic regulation in terms of timing and gene dosage for coordinated expression of their functions allowing the dissemination of SRIs and ACPs. The efficient activation of the machinery for DNA processing and mating pore assembly relies on the flexibility and accuracy of DNA binding by the activator complexes SetCD and AcaCD. For instance, SetCD can be tolerant to insertion of cargo DNA in the promoter driving expression of *traI* in SXT, an essential component of conjugal transfer (Poulin-Laprade et al., 2015).

#### Mechanism of Activation by SetCD and AcaCD

ChIP-exo experiments have revealed 11 SetCD-dependent promoters in SRIs and 19 AcaCD-dependant promoters in ACPs (Carraro et al., 2014a; Poulin-Laprade et al., 2015). SetCD- and AcaCD-dependent promoters have poorly conserved -10 and non-conserved -35 boxes, compared to the canonical σ^70^ promoter elements (Hawley and McClure, 1983; Kumar et al., 1993). In each promoter, the DNA motif recognized by the activator complex partially overlaps the -35 element, which is usually bound by the σ^70^ subunit of RNAP (Carraro et al., 2014a; Poulin-Laprade et al., 2015). This suggests that, as observed for FlhCD, SetCD and AcaCD compensate for the lack of a recognizable -35 elements by binding in the -35 region, facilitating the recruitment of σ^70^-bound RNAP to the promoters. As such, FlhCD, SetCD and AcaCD act as typical class II transcriptional activators (Browning and Busby, 2004). FlhCD was shown to interact with the C-terminal domain of RNAP (Liu et al., 1995). Biochemical characterizations are needed to establish whether AcaCD and SetCD directly interact with RNAP.

#### Activation of Integration, Excision, and Stability Functions

As SRIs maintain by integration in the host cell chromosome, the major contributor to their maintenance in a bacterial lineage is the integration/excision module (Hochhut and Waldor, 1999; Burrus and Waldor, 2003). This module contains the *int* gene coding for the integrase, a site-specific tyrosine recombinase, the *xis* gene coding for a recombination directionality factor, as well as their cognate attachment sites, i.e., *attP* on the circular form, or *attL* and *attR* at both ends of the chromosomally integrated SRI. Expression of both *int* and *xis* is SetCD-dependent, yet driven from two separate promoters (Burrus and Waldor, 2003; Poulin-Laprade et al., 2015). Stability of SRIs is also provided by toxin-antitoxin systems (TA) and a type II active partition system named *srpRMC* (SXT/R391 partition; Dziewit et al., 2007; Wozniak and Waldor, 2009; Carraro et al., 2015b). The *srpRM* genes code for the proteins driving the active partition of the excised element in daughter cells, while *srpC* is a centromere-like sequence bound by SrpR (Baxter and Funnell, 2014; Carraro et al., 2015b). Regulation of integration, excision and active partition of SRIs are interconnected as *srpRM* and *int* are cotranscribed from the same SetCD-dependent promoter (Poulin-Laprade et al., 2015).

As plasmids, ACPs maintain in bacterial lineages by autonomous replication, which is mediated by the *repA*/*oriV* locus in an AcaCD-independent fashion (Llanes et al., 1996; Carraro et al., 2014a). Orthologs of the SRI’s *srpRM* genes are also found in ACPs (*vcrx151*/*vcrx152* in pVCR94). Reminiscent of SRIs, expression of these *srpRM* orthologs is AcaCD-dependent (Carraro et al., 2014a). Interestingly, ACPs also carry genes coding for a type I ParABC-like partitioning system (Walker-type ATPase; *vcrx031*/*vcrx032* in pVCR94), whose regulation is likely independent of AcaCD (Baxter and Funnell, 2014; Carraro et al., 2014a, 2015b).

#### Activation of the Conjugative Machinery

SRIs and ACPs code for very similar conjugative machineries, as reflected by the syntenic organization of their transfer genes and the closely related proteins they encode (Fricke et al., 2009; Wozniak et al., 2009). The mobilization modules (*mob*) code for key factors involved in DNA molecule preparation (DNA processing functions) that will be translocated to the recipient cell through the type IV secretion system encoded by the *tra* modules. The relaxase TraI, with the help of the auxiliary protein MobI, is thought to recognize the origin of transfer (*oriT*) located immediately upstream of *mobI* in both SRIs and ACPs (Figures 1 and 3B,C; Ceccarelli et al., 2008; Carraro et al., 2014b). By analogy with other better characterized conjugative systems such as F, the resulting nucleoprotein complex, aka relaxosome, is thought to nick one DNA strand within *oriT* (Llosa et al., 2002). This DNA strand is delivered to the mating pore linking the donor and recipient cells. Based on the mechanism of single-stranded conjugative transfer of the F plasmid, it is assumed that SRIs and ACPs replicate using the rolling-circle mechanism during translocation of the transferred DNA strand. Several studies on ICEs from both Gram-negative and Gram-positive bacteria showed that ICEs are capable of intracellular rolling-circle plasmid-like replication (Kiewitz et al., 2000; Pembroke and Murphy, 2000; Dimopoulou et al., 2002; Grohmann, 2010; Lee et al., 2010; Carraro et al., 2011, 2015b; Sitkiewicz et al., 2011). This replication only occurs in a subpopulation of cells as it is conditional on element activation. Mechanistically, it does not strikingly differ from rolling-circle replication used for the stable maintenance of plasmids, uses *oriT* as an origin of replication and the relaxase TraI as a replication initiator protein. In fact, the replication module is part of the mobilization module (Grohmann, 2010; Lee et al., 2010; Carraro and Burrus, 2014). Although the exact mechanism remains to be elucidated, SRIs have been shown to replicate in an *oriT*, TraI and SetCD-dependent manner (Pembroke and Murphy, 2000; Carraro et al., 2015b).

Genome-wide footprinting of SetCD and AcaCD DNA binding coupled with transcriptomic analyses revealed that the syntenic *mob* modules of SRIs and ACPs are divided into different transcriptional units (Carraro et al., 2014a; Poulin-Laprade et al., 2015). In SRIs, SetCD binds upstream of *mobI* (*mob1*), *traI* (*mob2a*), and *traDJ* (*mob2b*). Interestingly, the canonic promoter driving the expression of *traI* is disrupted by an insertion into hotspot 5 (Poulin-Laprade et al., 2015). The -10 element of *P_traI_* is part of the conserved core and retained, while the -35 element is variable and provided by inserted cargo DNA. Alteration of *P_traI_* is associated with a poorer affinity for SetCD as determined by ChIP-exo, which could contribute to the lower transfer and replication of SXT compared with R391 (Carraro et al., 2015b; Poulin-Laprade et al., 2015). In ACPs, AcaCD activates the expression of *traIDJ* (*mob2*) from a unique promoter (Figure 1; Carraro et al., 2014a, 2015a). No ACPs available to date in the Genbank database harbor a *P_traI_* promoter altered by insertion of cargo DNA (Carraro et al., 2014a). Surprisingly, no AcaCD binding-site was detected upstream of *mobI*_A/C_ (formerly known as *vcrx001*, Carraro et al., 2014a,b). As for MobI of SRIs, MobI_A/C_ is essential for conjugative transfer of ACPs (Carraro et al., 2014b). The impact of such subtle differences on the regulation of conjugative transfer of SRIs and ACPs need to be experimentally addressed. Altered regulation of the *mob* functions can have drastic effects on the dynamics of these elements since initiation of transfer (*oriT* recognition and nicking by the relaxosome) was shown to be the rate limiting step of SRIs dissemination (Carraro et al., 2015b).

Other essential components for conjugative transfer of SRIs and ACPs are the pilus, which stabilizes the initial contact between cells, and the type IV secretion system (mating pore) through which DNA is translocated to recipient cells. This conjugative machinery is encoded by three mating pair formation modules (*mpf*) which are, as the *mob* modules, syntenic between SRIs, ACPs and the F plasmid (Figure 1; Lawley et al., 2003; Fricke et al., 2009; Wozniak et al., 2009). In both SRIs and ACPs, the *mpf1a* module contains the *traLEKB* genes, while *traAV* are found in *mpf1b* (Figure 1; Armshaw and Pembroke, 2013; Carraro et al., 2014a, 2015a; Poulin-Laprade et al., 2015). The *mpf2* modules are organized differently in SRIs (*mpf2*: *dsbC-traC-s093-trhF-traWUN*) and ACPs (*mpf2a*: *dsbC-traC-vcrx079-trhF-traW-vcrx082-traU* and *mpf2b*: *traN* in pVCR94ΔX), the latters expressing *traN* from its own AcaCD-dependent promoter (Figures 3B,C). Finally, the *mpf3* module (*traFHG*) has the same operon structure in both types of elements.

SetCD targets were exclusively found in the conserved backbone of SRIs (Poulin-Laprade et al., 2015). In contrast, AcaCD binding sites were also detected upstream of operons containing genes of unknown functions, as well as in regions that are not conserved (Carraro et al., 2014a, 2015a). The relevance of these AcaCD-regulated genes for the biology of ACPs remains to be determined.

#### Activation of RecA-independent Homologous Recombination Functions

In addition to conjugative transfer functions, SRIs and ACPs code for diverse mutagenic and recombination functions. Both types of elements include the well-conserved *bet* and *exo* genes, which code for a λ Red-like RecA-independent homologous recombination system (Garriss et al., 2009). This system contributes to the formation of hybrid ICEs by recombineering elements inserted in tandem in the chromosome, generating new patterns of antibiotic resistance genes. In both SRIs and in ACPs, the expression of *bet* and *exo* is under the control of the SetCD- and AcaCD-dependent *P_*s*089_* and *P_*vcrx*087_* promoters, respectively (Garriss et al., 2013; Carraro et al., 2014a; Poulin-Laprade et al., 2015). In both cases, the promoter driving their expression exhibits the highest ChIP-exo enrichment peaks. Although *bet* and *exo* are highly transcribed, their expression is hindered by a strong translational attenuator located upstream of *bet* in SXT (Garriss et al., 2013). This translational attenuator is also present in ACPs, but its functionality remains to be investigated.

## SetCD and AcaCD Trigger the Expression of Genomic Island-bound Genes

Several autonomous conjugative elements were shown to mobilize non-autonomous GIs using various mechanisms (Bellanger et al., 2014). For instance, the conjugative transposon Tn*916 trans*-mobilizes the 1.7 kb-GI mTnSAG1 from *Streptococcus agalactiae* by recognition of a cryptic *oriT* located within the *lnu*(C) gene, which confers resistance to lincomycin (Achard and Leclercq, 2007). ICEs from *Streptococcus thermophilus* were shown to *cis*-mobilize elements called CIMEs (*cis*-mobilizable elements) by a mechanism designated as accretion-mobilization (Pavlovic et al., 2004; Bellanger et al., 2011). SRIs and ACPs can also *trans*-mobilize diverse GIs using distinct strategies for their dissemination. Interestingly, these strategies are all coupled to the regulatory network of their cognate helper element (Daccord et al., 2010, 2012, 2013; Douard et al., 2010; Carraro et al., 2014a, 2015a; Poulin-Laprade et al., 2015).

### SRIs-dependent Mobilization of Genomic Islands

Characterization of the *oriT* sequence of SRIs allowed identification of chromosomal *oriT*-like sequences that were more than 63% identical (Ceccarelli et al., 2008; Daccord et al., 2010). Further investigations revealed that these cryptic *oriT* sequences belong to MGIs integrated at the 3′ end of *yicC* in the chromosome of *Vibrio*, *Alteromonas*, *Pseudoalteromonas*, and *Methylophaga* species (Daccord et al., 2010, 2013). The size of these MGIs ranges from 18 to 33 kb with a conserved core sequence of ∼5.5 kb encompassing four genes (*int_MGI_*, *cds4*, *cds8*, *rdfM*) and their cognate regulatory sequences. *int_MGI_* and *rdfM* code for the integrase and recombination directionality factor that allow MGIs to excise from and integrate into the host cell chromosome. Function of *cds4* and *cds8* remains unknown. The conserved backbone of MGIs is disrupted by DNA fragments that vary in size and gene content. Most of these genes code for adaptive functions such as type I and type III restriction-modification system that may confer resistance to bacteriophage infection (Daccord et al., 2013).

The initial step of an MGI mobilization by SRIs is its excision from the chromosome. Excision requires the transcriptional activation of *int_MGI_* and *rdfM* by the SRIs-encoded master activator SetCD (Figure 3B), and the subsequent recombination between the *attL_MGI_* and *attR_MGI_* attachment sites flanking the MGI (Daccord et al., 2012; Poulin-Laprade et al., 2015). The resulting circular extrachromosomal MGI carries *oriT*_MGI_, which acts as a *cis*-acting sequence that mimics the *oriT* of SRIs and hijacks the relaxosome encoded by SRIs. Ultimately, the MGI is translocated to the recipient cell throught the mating apparatus encoded by SRIs. Once in the recipient cell, the MGI becomes completely independent of the helper SRI and its transcriptional activator SetCD to establish itself in the new host. The MGI constitutively expresses *int_MGI_* at a low level, thereby allowing its own integration into the 3′ end of *yicC* (Daccord et al., 2012). MGI*Vfl*Ind1, initially isolated from *Vibrio vulnificus*, was used as a prototype to study MGIs and was reported to be mobilized at high frequency between *E. coli* strains by both ICE*Vfl*Ind1 and SXT (10^-3^ transconjugants per donor cell). This frequency rose to 10^–1^ transconjugants per donor cell upon overexpression of *setCD* or induction with mitomycin C treatment (Daccord et al., 2010, 2012). MGI*Vfl*Ind1 is also able to *cis*-mobilize over 1 Mb of chromosomal DNA located 5′ of *yicC* in an Hfr-like manner (Daccord et al., 2010). Chromosomal DNA mobilization by MGI*Vfl*Ind1 involves initiation of transfer at *oriT_MGI_* by the relaxosome of a SRI prior to excision of the MGI from the chromosome.

### ACP-dependent Mobilization of Genomic Islands

Discovery of the sequences targeted by AcaCD in ACPs was the cornerstone for the identification of potential chromosomal targets in genomes of several *Enterobacteriaceae* and *Vibrionaceae* (Carraro et al., 2014a, 2015a). Notably, multiple AcaCD binding sites were detected in the *Salmonella* genomic island 1 (SGI1), which confers MDR to pathogenic *S. enterica* and was reported to be mobilized in *trans* by ACPs by an unknown mechanism (Doublet et al., 2005; Douard et al., 2010; Carraro et al., 2014a). AcaCD sites were also detected in other GIs that are or could be mobilized by ACPs (Carraro et al., 2014a, 2015a).

#### SGI1-like Elements

SGI1 is a 43-kb chromosomal mobile element carrying a class 1 integron that confers resistance to ampicillin, chloramphenicol, streptomycin, sulfonamides and tetracycline (ACSSuT phenotype; Mulvey et al., 2006). SGI1 and related MDR-conferring GIs have been found integrated at the 3′ end of *thdF* (*trmE*) in a large variety of multidrug resistant *S. enterica* serovars and in *P. mirabilis* (Boyd et al., 2008; Wiesner et al., 2009; Hall, 2010; Girlich et al., 2015). All SGI1-like elements share a highly conserved ∼27 kb core region, which mostly contain genes of unknown function (Mulvey et al., 2006; Boyd et al., 2008). The conserved genes *int* and *xis* mediate SGI1’s excision from and integration into the chromosome (Doublet et al., 2005). Three conserved *tra* genes code for putative mating pore components sharing 40, 60, and 78% identity with ACP’s TraN, TraG and TraH, respectively. In most SGI1-like elements, the core region is disrupted between the resolvase-encoding gene *res* and *s044* by complex integrons conferring MDR (Boyd et al., 2008). Interestingly, a similar variable region is inserted in *s023* in the SGI1-variant SGI2 (formerly SGI1-J; Levings et al., 2008).

Currently, the genetic determinants and the nature of the interactions allowing the specific mobilization of SGI1 by ACPs remain largely unknown. Recent work revealed that the transcriptional activator AcaCD encoded by ACPs triggers the excision of SGI1 (Carraro et al., 2014a). Indeed, AcaCD-binding motifs were identified in the promoter region of the recombination directionality factor-encoding gene *xis* as well as upstream of putative regulatory and conjugation proteins (Figure 3C). SGI1 was reported to be highly stable, even after 350 generations without selective pressure (Kiss et al., 2012). Nevertheless, these assays were carried out in cells lacking an ACP. This is a major bias since SGI1 cannot excise in the absence of AcaCD (Carraro et al., 2014a). Interspecies mobilization of SGI1 between *S. enterica* and *E. coli* was reported to be highly variable (10^–5^ to 10^–2^ transconjugants per donor cell after overnight matings), depending on the *Salmonella* strain, the SGI1 variant and the IncA/C helper plasmid (Doublet et al., 2005; Kiss et al., 2012). In contrast, the frequency of transfer of SGI1 mobilized by pVCR94ΔX between *E. coli* strains was so high that virtually all recipient cells harbored SGI1 after mating (Carraro et al., 2014a).

#### MGI*Vmi*1-like Elements

ACPs also *trans*-mobilize MGI*Vmi*1, a 16.5 kb element that belongs to a family of MGIs unrelated to SGI1 and to the MGIs mobilized by SRIs (Carraro et al., 2014a, 2015a). AcaCD binding sites were detected upstream of *490* and *xis* (formally *420*), which code respectively for a 195-amino acids MobI-like protein and a 94-amino acid residue putative recombination directionality factor (Figure 3C). Similar characteristics are found in several GIs inserted in *Vibrio mimicus*, *Vibrio parahaemolyticus*, and *Shewanella putrefaciens* genomes (Carraro et al., 2015a). Although these GIs are highly variable in size and content, their conserved key features strongly suggest that they are mobilizable by ACPs. Excision of MGI*Vmi*1 was found to be AcaCD-dependent and its transfer requires the conjugative machinery encoded by ACPs. Here again, the exact mechanism remains unknown and further investigation is needed. However, based on the homology with the structure of the *mob1* mobilization module of SRIs and ACPs, we hypothesized that the *oriT* locus of these GIs lies within the large intergenic region located upstream of the AcaCD-dependent gene coding for a MobI homolog (Carraro et al., 2015a).

## Diversity of FlhCD-like Transcriptional Activators Amongst Conjugative Elements

**FIGURE 4:**
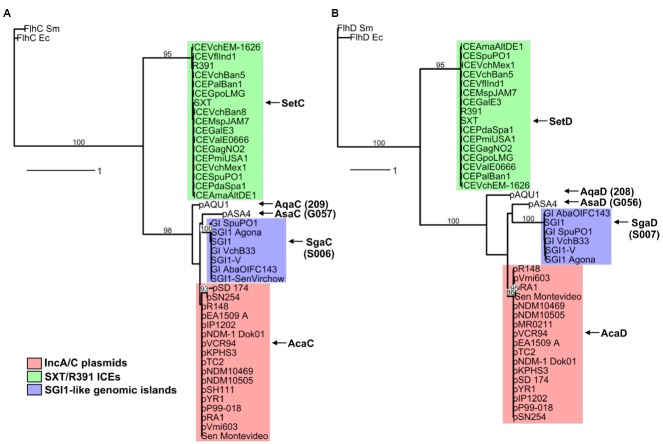
**Phylogenetic trees based on alignments of amino acid sequences of SetC/AcaC (A) and SetD/AcaD (B) orthologs.** The flagellar transcriptional activator proteins FlhC and FlhD of *E. coli* (Ec) and *Serratia marcescens* (Sm) were used as outgroups in phylogenetic analyses. Bootstrap values are indicated when over 80%. The individual scale bars represent genetic distances.

**FIGURE 5:**
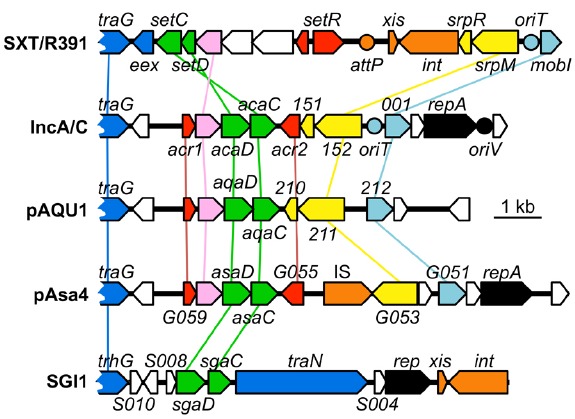
**Comparison of the genetic context of genes coding for SetCD/AcaCD orthologs in diverse mobile genetic elements.** Schematic representation of demonstrated or putative regulatory regions of SXT from *V. cholerae* O139 (AY055428.1), pVCR94 from *V. cholerae* O1 El Tor (NC_023291), pAQU1 from *P. damselae* subsp. *damselae* (NC_016983.1), pAsa4 from *Aeromonas salmonicida* (CP000645.1) and *Salmonella* genomic island 1 (SGI1) from *S. enterica* subsp. *enterica* serovar Typhimurium DT104 (AF261825.2). Arrows of similar color represent genes predicted to have similar functions and are color-coded as in Figure 1 legend. Circles indicate the position of origins of transfer (pale blue, *oriT*), of the origin of replication (black, *oriV*) of pVCR94 based on identity with pRA1, and the position of the *attP* site for chromosomal integration of SXT by site-specific recombination (orange).

A search for additional FlhCD-like regulators amongst other mobile genetic elements was carried out. Because homologies between SetCD and AcaCD, and the master flagellar activator FlhCD, are very weak, the Pfam HMM profiles for FlhC (PF05280) and FlhD (PF05247) domains are unsuitable to find functional orthologs of SetC/AcaC-like and SetD/AcaD-like activators in bacterial genomes. To solve this problem, we generated new HMM profiles based on alignments of the primary sequence of SetC/AcaC and SetD/AcaD protein orthologs. Screening of the Genbank non-redundant protein sequence database using these new profiles revealed a large number of homologous proteins encoded by diverse types of mobile genetic elements in *Enterobacteriaceae* and *Vibrionaceae*. Phylogenetic reconstructions using a representative subset of SetC/AcaC and SetD/AcaD orthologs revealed identical clustering in three distinct families distantly related to FlhC and FlhD: (i) SetC and SetD encoded by SRIs, (ii) AcaC and AcaD encoded by ACPs, (iii) putative proteins encoded by SGI1-like elements, S006 and S007, here renamed SgaC and SgaD (SGI1 activator subunits C and D), (iv) putative proteins encoded by pAQU1-like conjugative plasmids, 208 and 209 that we named AqaD and AqaC (pAQU1 activator subunits C and D), (v) putative proteins encoded by pAsa4, G057 and G056, here renamed AsaC and AsaD (pAsa4 activator subunits C and D; Figure 4). Interestingly, the genes coding for these putative transcriptional regulators are found in a similar genetic context in all cases (Figure 5). They are found in close proximity to a gene coding for a TraG homolog, a component of the conjugative apparatus, a gene coding for putative lysozyme-like protein. and genes coding for homologs of the SrpRM partition system and MobI protein. Further investigation are needed to confirm the functionality of these putative activator complexes regarding the activation of their cognate mobile GIs, and their potential impact on other genetic elements.

## Concluding Remarks and Perspectives

In the current context of rapid emergence and spread of MDR, it has become essential to get a better understanding of the dynamics and the mechanisms of transfer and regulation of mobile GIs that promote such resistance. A plethora of studies have been aimed at characterizing the determinants of transfer of various model conjugative genetic elements such as ICE*Bs1*, Tn*916*, CTnDOT, R388, pESBL, pSL20, R27 (Celli and Trieu-Cuot, 1998; Marra and Scott, 1999; Auchtung et al., 2005; Gibert et al., 2013; Waters and Salyers, 2013; Fernandez-Lopez et al., 2014; Yamaichi et al., 2015). Extensive research on SRIs and recent work on ACPs have refined our grasp of the biology and regulation of these major players of MDR propagation. Classical genetics and modern molecular methods have facilitated the complete characterization of the regulons of SetCD and AcaCD, which greatly improved our understanding of SRIs, ACPs, and the elements they mobilize.

Mobilization of GIs requires the SetCD- or AcaCD-dependent activation of their excision, and involves three distinct mechanisms of *oriT* recognition and DNA processing. First, the MGI-SRI model is based on the recognition of a MGI-borne sequence mimicking the *oriT* of the self-transmissible SRI. This *oriT* counterfeit is likely recognized as a genuine *oriT* by the relaxosome of SRIs, thereby leading to MGI transfer through the SRI-encoded mating pore. Second, the MGI*Vmi*1-ACP model likely relies on *oriT* recognition of MGI by its cognate MobI-like protein (MobI*_MGI_*), whose expression depends on AcaCD. The subsequent DNA processing at *oriT* of the MGI likely results from the interaction of MobI*_MGI_* with the MobI*_A/C_*-less relaxosome encoded by ACPs. Finally, the SGI1-ACP model remains the most elusive mechanism of mobilization as no *oriT_IncA/C_* sequence or MobI-like encoding gene has been identified so far in the sequence of SGI1-like elements. Further experiments are ongoing to precisely determine the mechanisms leading to the mobiliation of such GIs, and potentially of newly identified GIs.

Exploitation of experimentally determined SetCD and AcaCD recognition motifs to use them as specific tags for *in silico* analyses of bacterial genomes is a powerful approach to identify new mobile GIs mobilized by either SRIs or ACPs (Carraro et al., 2014a, 2015a). Moreover, additional FlhCD-like regulators were identified, which given their degree of divergence with AcaCD and SetCD, likely recognized unrelated DNA motifs. We anticipate that characterization of these motifs will facilitate the discovery of additional families of MGIs in sequenced bacterial genomes.

Mobile genetic elements and their bacterial host are inherently connected. Horizontally acquired DNA is often silenced by bacterial host factors such as H-NS-like proteins, most likely to limit the impact of exogenous DNA on endogenous regulatory networks and metabolic pathways (Dorman, 2004, 2014; Navarre et al., 2006; Singh et al., 2014). In the case of SRIs, IHF was shown to be mandatory for *V. cholerae* to act as a donor of SXT, but dispensable in *E. coli* donors (McLeod et al., 2006). The host factor Fis does not influence SXT transfer to or from *V. cholerae*. Further studies will be required to evaluate the influence of host factors on the dynamics of ACPs. Reciprocally, SRIs and ACPs, could interfere with the regulation of the host cell cellular processes. Besides the targets identified in MGIs, no clear interactions of SetCD and AcaCD with chromosomal loci in *E. coli* were detected (Carraro et al., 2014a; Poulin-Laprade et al., 2015). Nevertheless, considering the limitations of experimental settings and the broad host range of SRIs and ACPs, interactions with host metabolic pathways or other bacterial responses cannot be excluded. SetCD and AcaCD could also target plasmids of other incompatibility groups or other mobile GIs. Finally, other conjugative elements that code for FlhCD-like regulators could have a significant impact on their host biology.

Further investigations on the regulation of self-transmissible and mobilizable genetic elements will ultimately unravel the interconnections and the mechanism by which MDR and other adaptive traits spread among bacterial populations. In the war against the rampant emergence of untreatable pathogenic bacteria, fundamental knowledge regarding the key determinants at play for the dissemination of MDR will be an undeniable asset. To prevent a possible and imminent post-antibiotic era, mankind could find its salvation in the development of a new generation of molecules targeting key regulators aimed at confining or halting the exchange of MDR-conferring mobile genetic elements in patients, or even cure them from bacterial populations.

## Authors’ Note

After acceptance of this review, our results demonstrating the involvement of AcaCD in the excision of SGI1 (Carraro et al., 2014a) were confirmed by Kiss et al. (2015). This paper also explores the role of SgaCD (Figures 4 and 5 of this review) and strongly suggests that similarly to its IncA/C-encoded counterpart AcaCD, SgaCD of SGI1 activates the promoter of *xis* and the subsequent excision of this genomic island.

### Conflict of Interest Statement

The authors declare that the research was conducted in the absence of any commercial or financial relationships that could be construed as a potential conflict of interest.
